# Cobalt and Vitamin B12 in Dairy Cattle Nutrition: Requirements, Functions, and Interactions

**DOI:** 10.3390/ani15233477

**Published:** 2025-12-02

**Authors:** Martha Siregar, Gerald Salas-Solis, Antonio P. Faciola

**Affiliations:** Department of Animal Sciences, University of Florida, Gainesville, FL 32608, USA

**Keywords:** dairy cows, cobalamin, energy metabolism, propionate metabolism, ruminal microbiome, trace-minerals

## Abstract

Minerals are essential for ruminant health, productivity, and metabolic function, with trace minerals playing critical roles at narrow dietary margins. Cobalt (Co) is essential as it supports ruminal microbial synthesis of vitamin B12 (cobalamin), which acts as a crucial cofactor in energy and protein metabolism. This review summarizes the role of cobalt in dairy cattle nutrition, emphasizing its contribution to vitamin B12 synthesis, propionate metabolism, and milk production. Only 3–15% of dietary cobalt is converted to vitamin B12, and efficiency depends on dietary composition, forage-to-concentrate ratio, and ruminal microbiome. Deficiency leads to reduced intake, poor growth, anemia, reproductive failure, and decreased milk yield. Cow’s milk contains ~0.5 µg/L of vitamin B12, with greater concentrations in colostrum; diet composition, supplementation, and genetics contribute to variability. Current recommendations set the cobalt requirement at 0.2 mg/kg diet DM, yet multiple environmental and nutritional factors can restrict vitamin B12 synthesis. Limitations of this review include heterogeneity among the studies reviewed, such as differences in trial design, animal genetics, diet composition, and environmental conditions, which may introduce variability and affect the generalizability and consistency of the findings. Collectively, findings highlight cobalt’s pivotal role in supporting microbial activity, energy metabolism, and production outcomes in dairy cows.

## Simple Summary

Minerals are vital nutrients in dairy cattle diets because they support growth, health, reproduction, and milk production. Among these, cobalt is especially important because it allows rumen microorganisms to make vitamin B12, a vitamin that cows cannot produce on their own. Vitamin B12 is needed for energy production, glucose formation, and protein metabolism. When cows do not receive enough cobalt, vitamin B12 synthesis decreases, leading to poor growth, reduced appetite, lower milk yield, and health problems. Although only a small fraction of dietary cobalt is converted into vitamin B12, ensuring adequate supply is critical. The most recent guidelines recommend 0.2 mg cobalt per kg of diet dry matter (DM). This review summarizes the role of cobalt in ruminant nutrition, how diet composition and the rumen microbiome affect vitamin B12 synthesis, and how cobalt supplementation supports animal performance. Understanding these interactions can help improve mineral nutrition strategies and promote the health and productivity of dairy cattle.

## 1. Introduction

Minerals are essential nutrients in ruminant diets because they support growth, health, reproduction, and productivity. They function in bone development, enzyme activity, electrolyte balance, blood clotting, and immune responses [1]. Ruminant minerals are classified into macrominerals and trace minerals, based on the dietary concentration required to meet nutritional needs.

Among trace minerals, cobalt is indispensable because it supports ruminal microbial synthesis of vitamin B12 (cobalamin). Ruminants cannot produce this vitamin themselves, making cobalt supply critical. Vitamin B12 functions as a cofactor for two enzymes: methionine synthase, which catalyzes the conversion of homocysteine to methionine in amino acid metabolism, and methylmalonyl–CoA mutase, which contributes to the catabolism of specific fatty acids and amino acids. Adequate vitamin B12 also supports ruminal propionate production, which the liver converts to glucose, a major precursor for lactose synthesis and milk production in dairy cows [2].

The most recent NASEM guidelines [3] doubled the estimated cobalt requirement to 0.2 mg/kg of dietary DM. However, the cobalt concentration of forages and many feedstuffs is typically insufficient to meet microbial and animal needs, making supplementation necessary [4]. Common supplemental sources include cobalt carbonate, cobalt sulfate, cobalt chloride, and cobalt glucoheptonate, whereas cobaltous oxide is not recommended because of poor bioavailability [2,5]. In addition, some studies found that cobalt supplementation increases vitamin B12 in dairy cows [6], but other studies did not observe this effect. This variability is likely due to differences in study design, animal genetics, diet composition, and environmental factors. The objective of this review is to identify current knowledge gaps regarding vitamin B12 metabolism in dairy cows, and to provide a comprehensive synthesis of recent findings to guide future research and nutritional strategies. This review summarizes the role of cobalt in ruminant nutrition, with an emphasis on cobalt’s importance for vitamin B12 synthesis, its contribution to nutrient metabolism, and its effects on production outcomes in dairy cattle. Studies were included based on their relevance to cobalt and vitamin B12 metabolism in dairy cows; only peer-reviewed articles and reviews published in English were considered, while non-peer-reviewed sources, conference abstracts, and studies unrelated to dairy cattle were excluded.

## 2. Minerals for Ruminants

Minerals are essential nutrients in dairy cattle diets and are classified according to the concentration required by the animal. Macrominerals are required at percent levels of dietary dry matter, whereas trace minerals are required at parts per million (ppm) [5]. Essential macrominerals include calcium (Ca), phosphorus (P), magnesium (Mg), sulfur (S), sodium (Na), chloride (Cl), and potassium (K) [5]. These minerals have critical physiological roles, and supplementation is required when forages or rations are deficient. Failure to meet requirements can result in metabolic disorders or, at excessive concentrations, toxicity.

Trace minerals essential for dairy cattle include cobalt (Co), copper (Cu), iodine (I), manganese (Mn), selenium (Se), and zinc (Zn). Each contributes to key physiological processes, and both deficiency and excess can impair metabolic function. Deficiencies in dairy cattle are frequently linked to imbalances in trace-mineral supply. Maintaining trace-minerals within narrow optimal ranges is necessary to support growth, enzyme activity, cellular metabolism, and immune function [5,7,8]. Given the wide range of physiological roles and the narrow margin between deficiency and excess, a clear overview of essential minerals and their functions is valuable. Table 1 and Table 2 summarize the major macrominerals and trace minerals required by dairy cattle, highlighting their key physiological functions and the typical deficiency symptoms observed when dietary supply is inadequate.

## 3. Cobalt and Vitamin B12

Cobalt (Co) is an essential trace-mineral required in small concentrations, approximately 0.1 to 0.2 mg per kg of dry matter [3]. In ruminants, it is a vital as it facilitates the microbial production of vitamin B12 within the rumen [4]. Its importance was first identified in 1935 when it was discovered that cobalt supplementation could remedy a condition marked by loss of appetite and weight decline in ruminants [8]. A few years later, in 1948, it was found that cobalt was an essential component of vitamin B12 for sheep and cattle, and its lack caused conditions such as coastal disease (in sheep), wasting disease or enzootic marasmus in cattle [8]. Animals suffering from this deficiency commonly exhibit non-specific symptoms, including lower feed consumption, slowed growth, muscle wasting, coarse fur, and thickened skin. They also frequently had reproductive issues and a decline in milk production [9].

Ruminal microorganisms are not highly efficient at converting dietary cobalt (Co) into vitamin B12, with only about 3–13% of ingested cobalt being utilized for this process [10,11]. The production of cobalamin is influenced by factors such as dietary fiber and the overall intake of dry matter [12]. However, among all variables, the amount of cobalt in the diet remains the primary driver of vitamin B12 synthesis in the rumen [12,13]. If cobalt is lacking in the feed, the microorganisms’ ability to produce vitamin B12 drops sharply within just a few days [14]. Rumen microbiome engineering is an emerging research field focused on manipulating the ruminal microbial ecosystem to optimize fermentation processes and improve ruminant productivity. Techniques include dietary interventions, probiotic supplementation, and microbial community modulation to favor beneficial microorganisms involved in nutrient cycling and cobalamin production [15,16]. These interventions can aim to enhance the microbial synthesis of vitamin B12, improve feed efficiency, and reduce methane emissions, contributing to sustainable ruminant production [17]. Advances in metagenomics and microbial ecology have expanded our understanding of microbial interactions and pathways critical to rumen function, informing targeted strategies for microbiome engineering [15]. However, variability in host genetics, diet, and environment presents challenges for consistent outcomes, indicating the need for further research to develop precise and effective microbiome management approaches.

Efficient energy metabolism in ruminants depends on vitamin B12, which is synthesized by rumen microorganisms during fermentation, provided dietary cobalt is sufficient (above 0.5 mg/mL in ruminal fluid) [18]. If cobalt levels fall below this threshold, vitamin B12 synthesis is impaired, reducing its availability to the animal [8,12].

Ruminal microorganisms synthesize vitamin B12, but production may decrease with abrupt dietary changes or stress, and cobalt deficiency impairs absorption. Adequate dietary cobalt allows microorganisms to meet the vitamin B12 needs of the cow, supporting microbial growth and propionate metabolism essential for glucose production [2,7].

Vitamin B12 acts as a growth factor for ruminal microorganisms and is crucial for propionate production, a key precursor for glucose in ruminants [19,20]. Severe vitamin B12 deficiency impairs propionate utilization [21]. In microorganisms, vitamin B12 serves as a cofactor for enzymes like methylmalonyl-CoA mutase, involved in glucose formation, and tetrahydrofolate methyltransferase, which plays a role in methionine synthesis and methyl group transfer [7].

Ruminal microbial synthesis of vitamin B12 is influenced by several factors, including dietary cobalt levels, diet composition, and rumen microbial populations [13,22]. Dietary cobalt is essential for microbial synthesis of vitamin B12, with greater fiber diets promoting greater production compared to high-starch diets [23].

Ruminal microorganisms play a key role in vitamin B12 synthesis, though only a few specialized bacterial species actually produce it [24,25]. Studies have shown that species like *Selenomonas ruminantium* and *Megasphaera elsdenii* produce the greatest amounts of vitamin B12 [24,26]. A high ruminal vitamin B12 concentration correlates with greater abundance of *Prevotella*, while lower vitamin B12 levels associate with higher levels of *Bacteroidetes*, *Ruminiclostridium*, and *Butyrivibrio* [25]. This suggests that the composition of the rumen microbiome significantly influences vitamin B12 levels.

To better illustrate the relationship between cobalt and vitamin B12 in ruminants, Table 3 summarizes the key aspects, including requirements, functions, efficiency of utilization, deficiency symptoms, dietary and microbiome effects, and metabolic roles. This synthesis highlights cobalt’s central role in microbial vitamin B12 synthesis and its downstream effects on energy metabolism, growth, and production responses in dairy cattle.

## 4. Vitamin B12 Bioavailability

Particle size, chelation, and source type all affect the bioavailability of vitamin B12 in ruminants. In contrast to inorganic forms like cobalt carbonate, which have poorer solubility and ruminal utilization, organic sources of cobalt, such as cobalt acetate and cobalt lactate, often have higher water solubility and bioavailability [2,18]. We have found that organic sources of Cobalt, when compared to CoCO_3_, improve vitamin B12 synthesis [31]. Chelated mineral forms improve microbial access for effective vitamin B12 production by shielding cobalt from early interactions in the rumen. Particle size also affects the rate of dissolution; smaller particles have more surface area and are more bioavailable. Research on various cobalt sources and forms is still scarce, though, and results can vary based on animal characteristics, microbial populations, and diet composition [32,33].

## 5. Vitamin B12 in Milk

Cow’s milk contains approximately 0.5 µg/L of vitamin B12, while in colostrum, vitamin B12 concentration is 4 to 10 times greater than milk [29]. It has been shown that the composition and management of the diet in dairy herds can affect the concentration of vitamin B12 in milk, mainly because the ruminal synthesis of vitamin B12 is affected by the diet [30,34].

Genetic factors may partially account for the variability in vitamin B12 concentrations observed among cows [35]. Additionally, dietary supplementation with folic acid and vitamin B12, either individually or in combination, can alter folate and vitamin B12 concentrations in milk [36].

Ruminal microorganisms require cobalt and certain dietary conditions to efficiently synthesize vitamin B12, which is essential for the cow’s energy and protein metabolism [37]. Diets high in forage and fiber promote greater vitamin B12 production compared to high-starch diets, and the rumen microbiome composition also significantly influences vitamin B12 synthesis and its levels in the cow’s blood and milk [11]. Legumes such as alfalfa and clover serve as natural sources of dietary cobalt for ruminants in some regions [38].

To better illustrate the factors that affect vitamin B12 levels in milk, Table 4 summarizes key findings from previous studies. This table highlights the baseline differences between milk and colostrum, the role of diet composition and forage-to-concentrate ratio, as well as the effects of supplementation and genetic variability. Collectively, these factors demonstrate that both nutrition and genetics play roles in shaping vitamin B12 concentrations in milk and colostrum.

## 6. Cobalt Requirement in Dairy Cattle

Vitamin B12 requirements in ruminant diets are closely linked to cobalt needs, as cobalt is a component of the B12 molecule. Ruminal microorganisms require 0.07 to 0.11 mg cobalt per kg of feed for optimal function, or a dietary supply of 0.07 to 0.2 mg/kg [5,43].

However, the concentration of cobalt in the diet used for these processes is relatively low in cows, varying from 3% to 15% [12,13,44]. However, according to Girard et al. [18], only 4% of cobalt in the diet was used for the synthesis of vitamin B12.

Young ruminants, whose rumens are not yet fully functional, require vitamin B12 from their diet, such as from colostrum or milk, until their rumen matures around six to eight weeks of age [40]. Akins et al.,2013 reported that depending on weight and metabolic status, cattle need 1.2 to 2.4 mg of cobalt daily, with a dietary upper limit of 25 mg/kg dry matter [10]. Current recommendations specify cobalt requirements as 0.11 mg/kg dry matter because cobalt’s primary role is to support rumen microorganisms, whose cobalt needs vary with ruminal conditions [19,42].

Cobalt in the diet is absorbed as a cation and does not return to the rumen, rendering it unavailable to ruminal microorganisms. Most absorbed cobalt is excreted in urine, with smaller amounts lost in bile [32]. Recommended cobalt supplementation ranges from 0.11 to 0.35 mg/kg of feed dry matter, with a maximum safe level of 10 mg/kg; toxicity occurs at 30 mg/kg [35,41]. The NRC [7] recommends 0.11 mg/kg dry matter, supplying about 1.2 mg/day for dry cows and 2.4 mg/day for lactating cows, to maintain plasma vitamin B12 levels above 0.3 µg/L.

The recommended cobalt level in ruminant diets is about 0.2 mg/kg of dry matter, with supplementation typically ranging from 0.1 to 0.2 ppm [32]. However, adequate cobalt intake does not always guarantee sufficient vitamin B12 synthesis, as factors like season, feed cobalt content, grazing behavior, animal characteristics, diet composition, and soil contamination also influence vitamin B12 production and utilization [37,40,45].

The dietary requirements for cobalt and its role in vitamin B12 synthesis are highly interdependent. While only a small fraction of dietary cobalt is converted to vitamin B12, adequate supply is essential for optimal microbial activity and ruminant health. Table 5 summarizes the key aspects of cobalt utilization, daily requirements, supplementation ranges, and the factors that influence vitamin B12 synthesis efficiency.

While many studies demonstrate positive effects of cobalt or vitamin B12 supplementation on ruminant health and productivity, some research reports minimal or no significant responses; refs. [2,4,31] found that supplemental dietary cobalt did not significantly affect cobalt concentrations in milk, serum, or liver, nor improve metabolic status in dairy cows. Similarly, Tiffany et al. and Grace et al. [45,46] observed little improvement in growth performance or vitamin B12 status with cobalt supplementation in beef cattle. They reported inconsistent production responses in dairy cows to vitamin B12 supplementation, which were influenced by dietary and animal factors. More recently, Lopreiato et al. [47] found that maternal cobalt and folic acid supplementation combined with rumen-protected methionine had limited effects on offspring biomarkers. These mixed findings highlight the complexity of cobalt metabolism and vitamin B12 bioavailability in ruminants and emphasize the importance of considering dietary composition, animal genetics, and environmental factors when evaluating supplementation outcomes.

**Table 5:** Summary of Cobalt and Vitamin B12 requirements in ruminants.

**Aspect**	**Details**
Microbial requirement	0.07–0.11 mg Co/kg feed DM for efficient ruminal function [48]
Efficiency of dietary Co use	Only 3–15% of dietary Co used for vitamin B12 synthesis; ~4% reported by Girard and Matte, 2005) [36]
Young ruminants	Require dietary vitamin B12 directly until rumen is functional (6–8 weeks of age) [40]
Daily requirement	1.2–2.4 mg/day depending on weight and metabolic status [10]
Recommended dietary level	0.11–0.35 mg/kg DM [7]
Toxicity threshold	Toxic at ≥30 mg/kg DM [27]
Requirement updates	Sets requirement at 0.2 mg/kg DM, with 0.1–0.2 ppm supplementation adequate [3]
Factors influencing B12 synthesis	Season, forage type, concentrate/forage ratio, age, species, nutrient interactions, and soil contamination [40]

DM—Dry Matter, Co—Cobalt, NASEM—National Academies of Sciences, Engineering, and Medicine, NRC—National Research Council.

## 7. Conclusions

In conclusion, cobalt is an essential trace mineral for both ruminal microorganisms and ruminants. This is because cobalt is an essential component of vitamin B12 which is synthesized by ruminal microorganisms. Vitamin B12 is also an essential cofactor for optimizing energy metabolism in both ruminal microorganisms and ruminant animals. In ruminants, microbial utilization of cobalt for vitamin B12 synthesis is influenced by dietary cobalt levels, diet composition, and ruminal microbiome structure, affecting fermentation. The recommended cobalt supplementation is 0.2 mg/kg of dry matter, which enhances vitamin B12 synthesis and can improve ruminal fermentation and milk yield.

## Figures and Tables

**Table 1:** Essential macrominerals for dairy cattle: physiological functions and deficiency symptoms.

Mineral	Primary Physiological Functions	Deficiency Symptoms
Calcium (Ca)	Bone and teeth formation; muscle contraction; blood clotting	Rickets; milk fever; reduced growth
Phosphorus (P)	Bone and teeth formation; energy metabolism (ATP, phosphorylation)	Poor fertility; reduced appetite; rickets
Magnesium (Mg)	Enzyme cofactor; nerve transmission; muscle function	Grass tetany; muscle tremors
Sulfur (S)	Component of sulfur-containing amino acids (methionine, cysteine); microbial protein synthesis	Reduced microbial protein synthesis; poor growth
Sodium (Na)	Osmotic balance; nerve impulse transmission	Reduced appetite; poor growth; pica
Chloride (Cl)	Osmotic balance; gastric acid (HCl) Formation	Alkalosis; poor growth
Potassium (K)	Osmotic balance; acid–base regulation; muscle function	Muscle weakness; poor appetite; reduced milk yield

ATP—Adenosine triphosphate, HCL—Hydrochloric acid.

**Table 2:** Essential trace minerals for dairy cattle: physiological functions and deficiency symptoms.

Mineral	Primary Physiological Functions	Deficiency Symptoms
Cobalt (Co)	Required for vitamin B12 synthesis by ruminal microorganisms	Vitamin B12 deficiency; anemia; reduced appetite
Copper (Cu)	Enzyme cofactor; connective tissue and hemoglobin synthesis	Anemia; depigmentation; poor reproduction
Iodine (I)	Thyroid hormone synthesis; metabolic Regulation	Goiter; reproductive failure
Manganese (Mn)	Enzyme cofactor; bone formation; reproductive function	Skeletal deformities; poor reproduction
Selenium (Se)	Component of glutathione peroxidase; antioxidant defense	White muscle disease; retained placenta
Zinc (Zn)	Enzyme cofactor; wound healing; immune function	Parakeratosis; impaired wound healing; poor growth

**Table 3:** Relationship between Cobalt and Vitamin B12 in ruminants.

Aspect	Key Findings	References
Requirement	Cobalt required in very small amounts (~0.2 mg/kg DM). dietary supply often insufficient	[3,8,11]
Function	Essential precursor for microbial synthesis of vitamin B12 (cobalamin) in the rumen	[27]
Efficiency	Conversion of dietary Co to vitamin B12 is low (3–13% of the dietary cobalt intake)	[18,28]
Threshold for synthesis	Ruminal synthesis requires >0.5 mg/L Co in ruminal fluid	[9,12,19]
Deficiency signs	Reduced intake, poor growth, muscle wasting, rough coat, reduced milk yield, reproductive issues	[9,20]
Metabolic role of B12	Cofactor for methylmalonyl-CoA mutase (gluconeogenesis via propionate) and methionine synthase (methyl transfer, protein metabolism)	[7,22]
Dietary effects	High-fiber diets increase vitamin B12 synthesis (up to 3× greater vs. high-starch diets); starch depresses synthesis	[10,29]
Microbiome effects	Only a few microorganisms synthesize B12: Selenomonas ruminantium, Megasphaera elsdenii, Butyrivibrio fibrisolvens; greater B12 linked to Prevotella	[22,25,30]

DM—Dry Matter, Co—Cobalt.

**Table 4:** Factors affecting Vitamin B12 concentration in milk.

Factor	Effect on Vitamin B12 in Milk	Reference(s)
Baseline levels	Milk contains ~0.5 µg/L vitamin B12; colostrum 4–10× greater	[39]
Diet composition	Affects ruminal synthesis → alters milk vitamin B12	[40,41]
Genetics	Explains part of cow-to-cow variability	[35,40]
Folic acid and B12 supplementation	Increases folate and vitamin B12 concentrations in milk	[42]
Forage/concentrate ratio	60:40 diet → greater ruminal synthesis of active vitamin B12 than 40:60	[37]
Dietary fiber	Positive correlation with milk vitamin B12 concentration	[40]
Dietary crude protein	Negative correlation with milk vitamin B12 concentration	[40]
Natural cobalt sources	Legumes (alfalfa, clover) provide cobalt for rumen B12 synthesis	[10]

## Data Availability

No new data were created or analyzed in this study. Data sharing is not applicable to this article.
